# Dr. Charles Sabin Taft

**Published:** 1901-01

**Authors:** 


### OBITUARY NOTES.

Dr. Charles Sabin Taft, of Mount Vernon, N.Y., died at his residence in that city December 18, 1900, aged 65 years. Dr. Taft was an acting assistant surgeon, U. S. Army, at the time President Lincoln was assassinated, April 14, 1865, and, being in Ford's theatre at the time, was the first physician to render professional service to the dying president. After the war Dr. Taft located at Washington, where he established a high-class drug store, but later resided at Mount Vernon, where he died. He was a native of Lyons, Wayne County, where he was born in 1835.

